# Internalized stigma in mental health staff with lived experience of mental crises–Does the professional role protect against self-stigmatization?

**DOI:** 10.3389/fpsyt.2022.1078478

**Published:** 2023-01-12

**Authors:** Stefan Stuetzle, Anna Brieger, Christian Lust, Angel Ponew, Sven Speerforck, Sebastian von Peter

**Affiliations:** ^1^Evangelische Hochschule Dresden, University of Applied Sciences for Social Work, Education and Care, Dresden, Germany; ^2^Department of Psychiatry and Psychotherapy, Medical University Brandenburg Theodor Fontane, Neuruppin, Germany; ^3^Department of Psychiatry and Psychotherapy, University of Leipzig Medical Center, Leipzig, Germany

**Keywords:** stigma, mental illness, self-stigmatization, lived experience, staff mental health

## Abstract

**Objective:**

The stigma of mental illness is widespread in the general population and also among healthcare and psychiatric professionals. Yet, research on the self-stigma of the latter is still limited. The purpose of this article was to assess self-stigma and its correlates in mental health professionals with lived experiences of mental crisis and treatment.

**Methods:**

In a cross-sectional exploratory research project, 182 mental health professionals with lived experiences of mental crisis and treatment from 18 psychiatric hospital departments in the German federal states of Berlin and Brandenburg were surveyed on their lived experiences, self-stigma, perceived stigma in the workplace, subjective vulnerability to crises, and meaningfulness of lived experiences. To investigate the relationships between the variables, manifest and latent correlation analyses were calculated.

**Results:**

Results showed low levels of self-stigma and perceived public stigma in the workplace. Self-stigma was significantly and positively associated with workplace stigma and subjective vulnerability to crisis, but not with identification with lived experiences.

**Conclusion:**

The relationship between self-stigma, workplace stigma, and vulnerability should be investigated in terms of mutual causality in order to derive possible strategies of reducing self-stigma along with its detrimental effects. Possible reasons for the low levels of self-stigma are discussed in the light of limitations, including processes of self-selection, with highly self-stigmatizing individuals being possibly discouraged from participating. Strategies to enhance sampling quality are briefly discussed.

## 1. Introduction

The stigma of mental illness is still widespread in the general population (1–3) and among healthcare and psychiatric professionals (4–6), including nurses (7) and physicians (8). Stigma has been identified as a means of exercising power over people, thereby keeping them “down, in, or out” (9). Consequently, individuals affected by stigmatization are faced with multiple forms of discrimination, like social exclusion or disadvantages in the job or housing market (10). Moreover, people who experience stigmatization tend to internalize stigmatizing attitudes, a phenomenon known as self-stigma (11).

Health professionals usually regard themselves as rather strong and invulnerable (12, 13). However, a small but growing body of research suggests that experiences of mental problems are widespread among mental health staff (5, 14, 15). Mental health workers with lived experiences of mental health problems–sometimes referred to as “wounded healers” (16)–are faced with stigmatization themselves, a topic that is still underexplored (17–19). Presumably, the lack of discussion of these phenomena is due to silencing, or the unwritten rules of health care organizations which discourage staff from disclosing instances of mental health issues, for example by ignoring, disregarding, or dismissing mental health workers’ reports of mental problems (20, 21). Such experiences may make it harder for mental health professionals to integrate experiences of mental crises or disorders into their identity. A qualitative study demonstrated that mental health professionals with lived experiences of mental health service utilization constructed and switched between two different and largely unintegrated identities (“professional” and “patient”) (22).

Mental health professionals’ ways of coping with their lived experiences and the associated stigma not only affect their own wellbeing but also influence their approach to the users of their services. Several studies showed that mental health professionals with lived experiences of mental illness used these experiences to deepen their empathic understanding of their clients, leading to enhanced therapeutic relationships (17, 19, 23, 24).

Despite a certain interest in mental health professionals’ lived experiences in the last years (25), the question of self-stigma (11) has not been addressed in this context. The goal of the current study was to better understand if and to what extent mental health professionals with lived experiences of mental crisis or treatment hold self-stigmatizing attitudes, and how these attitudes are interrelated with other aspects associated with stigmatized identities, namely public stigma in the workplace, vulnerability to mental crises, and identification with lived experiences.

As self-stigma is induced and maintained by public stigma (26, 27), a positive association between participants’ levels of self-stigma and their perception of stigmatizing attitudes toward individuals with mental health problems in their colleagues was expected. Further, a person’s self-perception as vulnerable to mental crisis may contribute to the process of self-stigmatization by enhancing the application of the common stereotype of mental instability or weakness (27, 28) to oneself. Therefore, participants with higher levels of perceived vulnerability were presumed to report higher self-stigma. As to stigmatized individuals’ identification with their stigmatized attributes, previous research has shown divergent associations with stigma and self-stigma, respectively. On the one hand, the centrality of a stigmatized identity, or the meaning it has for one’s self-definition, can increase detrimental effects of stigmatization in persons with concealable stigmata (29). On the other hand, the centrality of a stigmatized identity can decrease self-stigma in individuals with lived experience of mental distress (30). Also, persons who are diagnosed with schizophrenia can diminish self-stigmatizing attitudes by accepting their diagnosed condition as an unalterable part of their life (31). Hence, in the current study, it was hypothesized that a higher identification with one’s lived experience was associated with less self-stigmatization.

## 2. Materials and methods

### 2.1. Sampling

The current study presents results of a mixed-method research project that explored mental health professionals’ approach to their lived experiences of mental crisis and treatment. The research was based on a number of dissertation projects located at the Medical School Brandenburg and in cooperation with the University of Leipzig Medical Center. The research group consisted of researchers with and without lived crisis experiences, enabling the research staff to use diverse forms of knowledge and to discuss different perspectives concerning the research questions, results, and interpretations. Drawing on emancipatory models of peer support, i.e., the employment of people with personal experiences of mental health problems in mental health care (32), crisis experience was considered a gain (not deficit) that may enhance patient care.

Subsequent to a qualitative research phase, an online survey was conducted between May and September 2020 (33). In order to obtain a representative data basis, all 33 psychiatric hospital departments in the German federal states of Berlin and Brandenburg were addressed. Digital invitations were sent to management staff (head physicians, nursing management, management of therapeutic services) and distributed to all employees directly engaged in patient care. The participation in the survey was voluntary and anonymous. A total of 215 mental health professionals from 18 psychiatric hospital departments took part in the survey, representing all relevant professional groups. From these, 182 participants (or 84.7%) indicated that they had lived experiences of mental crisis (defined as episode of psychological strain and/or impairment of functioning, from single stressful events up to and including longer lasting clinical pictures) and/or treatment. These 182 participants constituted the sample of the current study.

### 2.2. Measures

The following measures were employed. When necessary, item wordings were adapted for the purpose of the current study (e.g., “mental crisis” instead of “mental illness”). All measures were rated on a 5-point Likert scale (0 = *I totally disagree*, 4 = *I totally agree*).

#### 2.2.1. Self-stigma

To gauge the level of self-stigma, participants were presented the ISMI-9 scale (34). This instrument was developed from the Internalized Stigma of Mental Illness (ISMI) scale (35). This instrument measures internalized or self-stigma, defined as the devaluation, withdrawal, and shame caused by applying negative stereotypes to oneself (36). To the authors’ knowledge, the ISMI-9 scale was never used before to assess self-stigma in mental health professionals. The German translation of the ISMI-9 items used in the current study (e. g., “Negative stereotypes about mental illness keep me isolated from the ‘normal’ world”) was adopted from a validation study by Sibitz et al. (37).

#### 2.2.2. Perceived workplace stigma

To measure perceived workplace stigma, the participants were presented items derived from the Perceived Devaluation-Discrimination (PDD) scale which is based on the modified labeling theory of stigma (38, 39). The PDD scale originally comprises twelve items and measures perceived stigmatizing attitudes toward individuals with mental illness in a given environment. Four items were formulated based on original items from the PDD according to the purposes of the current study (e.g., “Most of my colleagues would accept a person with lived crises/treatment experiences as a co-worker”).

#### 2.2.3. Vulnerability to crises

Vulnerability to crises was measured using the Self-Identification of Mental Illness (SELF-I) scale (40, 41). This instrument consists of five items and assesses the degree to which given symptoms are interpreted as indicators of a mental illness (e.g., “I am the type of person that could be prone to having a mental crisis”). As one item refers to current symptoms, it was omitted from the survey.

#### 2.2.4. Identification with crisis experiences

To asses participants’ identification with their lived experiences, a single item was used. Participants were asked to what degree they regarded their crisis experiences as meaningful for their personal identity formation (“My experience of crisis/treatment is an important part of my identity”). As social psychological research indicates, identification can be measured validly by using a single item (42).

### 2.3. Statistical analyses

The study variables were explored using descriptive statistics. To examine the factor structures of the employed scales, confirmatory and exploratory factor analyses (CFA and EFA, respectively) were conducted. Scale reliability was evaluated by testing the internal consistency using Cronbach’s alpha. The relationships between the ISMI-9 scale on the one hand and perceived stigma, vulnerability, and lived experience identification on the other hand were explored *via* manifest correlation analyses. Additionally, latent correlations were calculated *via* structural equation modeling (SEM) in order to counter potential estimation problems due to the low reliability of the SELF-I scale. Multivariate normality, a prerequisite for the use of SEM (including CFA), was tested using Mardia’s normalized estimates of multivariate kurtosis and skewness (43) which revealed non-normality. Therefore, SEM analyses were conducted using bootstrap calculations. All statistical analyses were conducted with STATA (“STATA,” 2021).

## 3. Results

Descriptive analyses of all items used in the current study revealed acceptable values. One item from the ISMI-9 had a kurtosis of 11.4, slightly over the conventional threshold (44).

### 3.1. Sample

Data were collected from 182 mental health professionals. The sample was 74.7% female, 24.2% male, and 0.6% diverse, with an average age of 42.3 years old. Participants were 33.5% nurses, 25.3% physicians, 20.9% psychologists, 6.0% social workers, and 14.3% special therapists and other professions (see Table 1).

**TABLE 1:** Sample characteristics.

		Female		Male		Diverse			
Profession	*N*	*n*	%	*n*	%	*n*	*%*	*M*_Age_	*SD*
Total	182	136	74.7	44	24.2	1	0.6	42.3	10.6
Social workers	11	10	90.9	–	–	1	9.1	47.5	8.6
Nurses	61	42	68.9	19	31.2	–	–	42.6	10.2
Psychologists	38	31	81.6	7	18.4	–	–	37.7	9.6
Physicians	46	30	65.2	15	32.6	–	–	41.5	10.6
Special therapists and miscellaneous	26	23	88.5	3	11.5	–	–	47.3	10.9

One participant did not indicate a gender; three participants did not indicate their age.

### 3.2. Scales

#### 3.2.1. Self-stigma

A CFA of the ISMI-9 with one latent factor revealed a rather unsatisfactory model fit, χ^2^ = 68.56, df = 27, *p* < 0.001, CFI = 0.89, RMSEA = 0.09, 90% KI [0.07,0.12], SRMR = 0.06. A second CFA was conducted drawing on the original factor structure of the ISMI-29. Four of the original subscale domains were modeled as covarying latent factors (namely, *alienation*, *social withdrawal*, *stereotype endorsement*, and *stigma resistance*), with two items loading on each factor. As the fifth subscale *(discrimination experience)* is represented only with a single item in the ISMI-9, it could not be integrated as factor due to the consequent problems concerning model identification. This model showed higher fit (see Supplementary Figure 1).

To further clarify the factor structure, an EFA was conducted. A principal axis factor analysis with oblique rotation (direct oblimin) revealed a factor with an Eigenvalue of 2.90, explaining 98.8% of the total variance. This seeming contradiction between the results of the CFA and EFA, respectively, is in line with the purpose of the ISMI-9, that is to represent the various facets of self-stigma while being statistically one-dimensional (34). Therefore, the ISMI-9 was considered as one-factorial construct for the purposes of the current study, and the nine items were combined into one scale calculating the mean. The internal consistency of the scale was acceptable (α = 0.80; see Table 2).

**TABLE 2:** Psychometric properties of study variables.

Variable	*M*	*SD*	α	Range	Skewness	Kurtosis
Self-stigma (ISMI-9)	0.61	0.52	0.80	0–2.22	1.02	3.54
Workplace/Public stigma in the workplace	0.88	0.74	0.87	0–3.75	1.05	4.16
Vulnerability (SELF-I)	1.94	0.74	0.68	0.25−4.00	0.35	3.09
Identification with lived experience	2.91	1.08	−	0−4.00	−1.04	3.64

*N* = 182. Potential range of all variables: 0–4.00.

The average self-stigma proved to be rather low in the current sample (*M* = 0.61, *SD* = 0.52), therefore a more detailed *post-hoc* look was taken. Analogous to the procedure of Brohan et al. (45), the ISMI-values were broken down into four categories: <1 minimal, 1–2 low, 2–3 moderate, and >3 high self-stigma. Over three quarters of participants (140, or 76.9%) indicated minimal self-stigma, 37 (20.3%) participants rated low self-stigma, and 5 (2.8%) reported moderate self-stigma. No participants reached high levels of self-stigma.

#### 3.2.2. Workplace stigma

A CFA of the four items representing workplace stigma with one latent factor showed an acceptable model fit (χ^2^ = 6.25, df = 2, *p* = 0.044, CFI = 0.99, RMSEA = 0.11, 90% KI [0.02,0.21], SRMR = 0.02), and a reliability analysis revealed a suitable internal consistency (α = 0.87). A scale was built by calculating the mean. With *M* = 0.88 (*SD* = 0.74), participants’ average perception of stigmatizing attitudes at their workplace was relatively low. Over 75% of the sample indicated workplace stigma levels below the midpoint of the scale (2.0).

#### 3.2.3. Vulnerability to crises

Vulnerability to mental crises, as measured by the SELF-I scale, is considered to be a one-dimensional construct (40). However, a CFA with one latent factor showed poor model fit, χ^2^ = 18.46, df = 2, *p* < 0.001, CFI = 0.89, RMSEA = 0.21, 90% KI [0.13,0.31], SRMR = 0.08. An EFA (principal axis factor analysis) was conducted on the four items. One of the extracted factors met Kaiser’s criterion (46), suggesting one factor. However, the scree plot was ambiguous, and a parallel analysis suggested two factors. An oblique rotation (direct oblimin) produced a factor solution with all items loading on a single factor with at least λ = 0.29, and this factor explained 76.7% of the variance. As a result, the four items were combined into one scale by calculating the mean. The internal consistency was α = 0.68, just under the commonly accepted threshold of 0.70 (47). Participants indicated an average vulnerability of *M* = 1.94 (*SD* = 0.74).

#### 3.2.4. Identification with crisis experiences

On average, participants rated their identification with crisis experiences as relatively high, *M* = 2.91 (*SD* = 1.08) which indicates that participants rather accept their crisis experiences as an important part of their identity.

### 3.3. Interrelations between the study variables

As shown in Table 3, the ISMI-9 scale was correlated significantly and positively with perceived stigma and vulnerability, whereas the negative correlation with the identification with lived experience clearly failed to reach a level of significance (*r* = −0.10, *p* = 0.187).

**TABLE 3:** Correlations between study variables.

	Variable	1	2	3	4	5	6
1	Self-stigma (ISMI-9)	1					
2	Perceived stigma	0.36***	1				
3	Vulnerability (SELF-I)	0.34***	0.01	1			
4	Identification with lived experience	-0.10	-0.05	0.03	1		
5	Age^a^	-0.03	0.04	-0.08	0.16*	1	
6	Sex^bc^	0.03	-0.03	-0.10	-0.17*	-0.05	1

*N* = 182.

^a^*n* = 179.

^b^*n* = 181. ^c^0 = female, 1 = male, 2 = diverse.

*p < 0.05, ***p < 0.001.

The results of the latent correlation analysis *via* SEM (see Supplementary Figure 2) corresponded to the results of the manifest correlation analysis The ISMI-9 scale showed substantial positive correlations with perceived stigma and vulnerability, and a negative but non-significant correlation with lived experience identification.

## 4. Discussion

The current study aimed to assess the level of self-stigma among mental health workers with lived experiences of mental crisis or treatment, and to investigate its relationships with other relevant phenomena, namely perceived stigma in the workplace, subjective vulnerability to mental crises, and identification with the lived experience. The results indicate that mental health workers with lived crisis experience show only minimal to low levels of self-stigma. As expected, self-stigma was positively and significantly correlated with stigma in the workplace and subjective vulnerability to mental crises, whereas the workplace stigma and subjective vulnerability were not interrelated.

### 4.1. Self-stigma–Not a problem for mental health professionals with lived experiences?

The low levels of self-stigma among the surveyed mental health professionals are a rather surprising result, regarding the high stigma toward mental problems that is still prevalent both in general society (2) and the mental health system (8, 48). Further, experiences of mental crisis and treatment are described to be incompatible with (mental) health workers’ typical self-concept of strength and invulnerability (12, 13), also guided by rather implicit rules that discourages professionals from disclosing their mental health problems (5, 20, 21).

A possible reason for the strikingly low levels of self-stigma may be found in the rather wide operationalization of lived experience employed in the current study: Explicitly including any single crisis episode, this operationalization may have provoked less self-stigmatization compared to one that would have used, for instance, DSM criteria as a cut-off for participants’ self-assigning of a mental crisis. Furthermore, a growing body of evidence shows substantial variance in stigma across specific diagnoses, with schizophrenia and substance abuse being more stigmatized than, for example, depression (1). On the other hand, the experiences reported by the participants in the online survey–at least in part–were rather severe, as presented in the main publication on our survey (33): A substantial percentage of the sample reported instances of suicidal ideation and the use of medication to overcome crises, which they self-categorized under almost all DSM categories, including psychotic and personality disorders. Further, most of the participants used mental health care treatment, including licensed psychiatrists and psychiatric hospitals. Facing these rather severe experiences of mental crisis and treatment, it remains surprising that only a small fraction of the participants indicated moderate levels of self-stigma, and none indicated high levels of self-stigma.

Instead, the surprisingly low levels of self-stigma may have been influenced indirectly by this rather wide crisis definition: As shown in the main publication on our survey, despite their rather severe experiences, our participants did not identify with their patients (33). On the contrary, they seemed to need a clear psychological distance from their patients and other crisis experienced colleagues. This need for distance was even stronger in participants who showed higher rates of personal identification with their crisis experiences. In short, these results show that own crisis experiences, even of rather severe nature, do not lead to an identification as “mentally ill” in the surveyed staff. Consequently, this lack of identification prevents self-stigmatization.

Another psychological mechanism that may explain the staff’s low self-stigma is that high levels of identification with one’s own lived experiences may lead to higher self-acceptance, which in turn may diminish self-devaluation and protect from (self-)stigmatization (31). Yet, in the current study the expected negative relationship between self-stigma and identification with lived experience did not prove statistically significant, providing no evidence for this mechanism. It is possible that the statistical non-significance of the correlation was due to the low variance of the ISMI-9 scale which can lead to the underestimation of covariance-based measures (like correlations). Also, the salience of the stigmatized crisis and/or treatment experiences may be of relevance (29, 49), with higher salience presumably being associated with higher levels of self-stigmatization. It is unclear to what extent mental health professionals with own experiences of mental distress think about these experiences in their everyday working life. Therefore, the role of the salience of lived experiences in this context should be further investigated.

Moreover, the time of occurrence of the crisis and individual strategies of stigma coping may also be of relevance in explaining the low levels of self-stigma in the current sample. Stigmatized individuals use different strategies to manage the detrimental effects of stigma, e.g., cognitive separation from the stigmatized group (50) or the refusal to apply a stereotype to oneself (51). Crises that may have been severe but have occurred a rather long time ago may be more effectively processed and hence less relevant for the self-concept today in comparison to current or rather recent crises, resulting in less self-stigma. Associations between self-stigma and the time of occurrence of participants’ crisis experiences could not be explored, as we did not collect data on the latter.

A further explanation could be a self-selection bias: Almost 85% of the participants in the current study reported lived experiences of mental crisis or treatment, which is about twice the lifetime prevalence of mental disorders in Germany of 42% (52). It is possible that mental health professionals with lived experience had shown a higher interest in the topic of the study, and hence a higher readiness to participate, especially if they did not feel ashamed of their experiences, for instance in case of not classifying them as an illness. On the other side of the spectrum, individuals with high self-stigma (maybe feeling “really” mentally ill) may have felt unsettled or ashamed when being confronted with the topic of the study and therefore abstained from participating.

### 4.2. Workplace environment, self-stigma, and vulnerability–a vicious circle?

Previous cross-sectional studies (mostly validation studies) show that individuals with high self-stigma perceive their environment as more stigmatizing (37, 53). In the current study, self-stigma was also positively correlated to perceived workplace stigma, as well as to subjective vulnerability to mental crisis. Due to the cross-sectional design, these results cannot by interpreted as causal relations.

Thus, the question arises if a more pronounced self-stigma leads to higher salience of stigmatizing environmental cues (higher perceived stigma), thereby weakening the feeling of mental stability(higher vulnerability)? Or, conversely, does the exposure to a more stigmatizing workplace environment cause higher salience of one’s crisis experiences, leading to higher self-stigma which increases subjective vulnerability? Maybe both explanations are in effect, both constituting a vicious circle. To shed light on the causality between self-stigma, workplace stigma, and vulnerability, these relationships should be investigated in prospective research designs, thereby introducing possible strategies to reduce the harmful effects of self-stigma.

### 4.3. Limitations

Due to the unrepresentative sample and the cross-sectional design, the current study is limited in its explanatory power. Also, all data is based on self-reports and therefore may be distorted by common method bias (54). Further, as mentioned before, the definition of the crisis variable was rather wide, leaving almost no possibility to not qualify as crisis-experienced. Yet, after thorough discussion in our research group, we failed to find a construct with a higher threshold that did not reproduce the stigmatization of mental illness. Moreover, the psychometric properties of the ISMI-9 scale were not yet assessed in a representative sample of (mental) health professionals, leaving its validity for our study sample unclear.

### 4.4. Conclusion

The current study aims to contribute to the exploration and ultimate removal of the taboo of the “wounded healers” (16), i.e. (mental) health professionals with own lived experiences. To the authors‘ knowledge, this is the first study to use the ISMI-9 scale to explore self-stigma in mental health workers with lived experience of mental crisis. The reported levels of self-stigma were surprisingly low, suggesting a self-selected study sample. To reduce self-selection bias and to obtain representative survey samples, awareness for the topic of health professionals with lived experience should be raised among psychiatric hospital management. Also, study participation rates in this field might be elevated by creating incentives for potential participants. Additionally, recruitment processes beyond organizations (e.g., *via* mandatory professional associations) should be considered.

## Data availability statement

The raw data supporting the conclusions of this article will be made available by the authors, without undue reservation.

## Ethics statement

Ethical review and approval was not required for the study on human participants in accordance with the local legislation and institutional requirements. Written informed consent for participation was not required for this study in accordance with the national legislation and the institutional requirements.

## Author contributions

SSt performed the statistical analysis and wrote the first draft of the manuscript. SPe wrote sections of the manuscript. All authors contributed to the conception and design of the study, manuscript revision, read, and approved the submitted version.
