# Supplementary material for: Internalized stigma in mental health staff with lived experience of mental crises–Does the professional role protect against self-stigmatization?
Source: Front Psychiatry. 2023 Jan 12;13:1078478. doi: 10.3389/fpsyt.2022.1078478 (PMC9877507; doi:10.3389/fpsyt.2022.1078478)
Supplement: Supplementary file 1 [file Presentation_1.PDF]

Supplementary Material

Internalized Stigma in Mental Health Staff With Lived Experience of Mental Crises – Does the Professional Role Protect Against Self-stigmatization?

Stefan Stuetzle*, Anna Brieger, Christian Lust, Angel Ponew, Sven Speerforck, Sebastian von Peter

*** Correspondence:** Stefan Stuetzle: stefan.stuetzle@ehs-dresden.de

Supplementary Figures

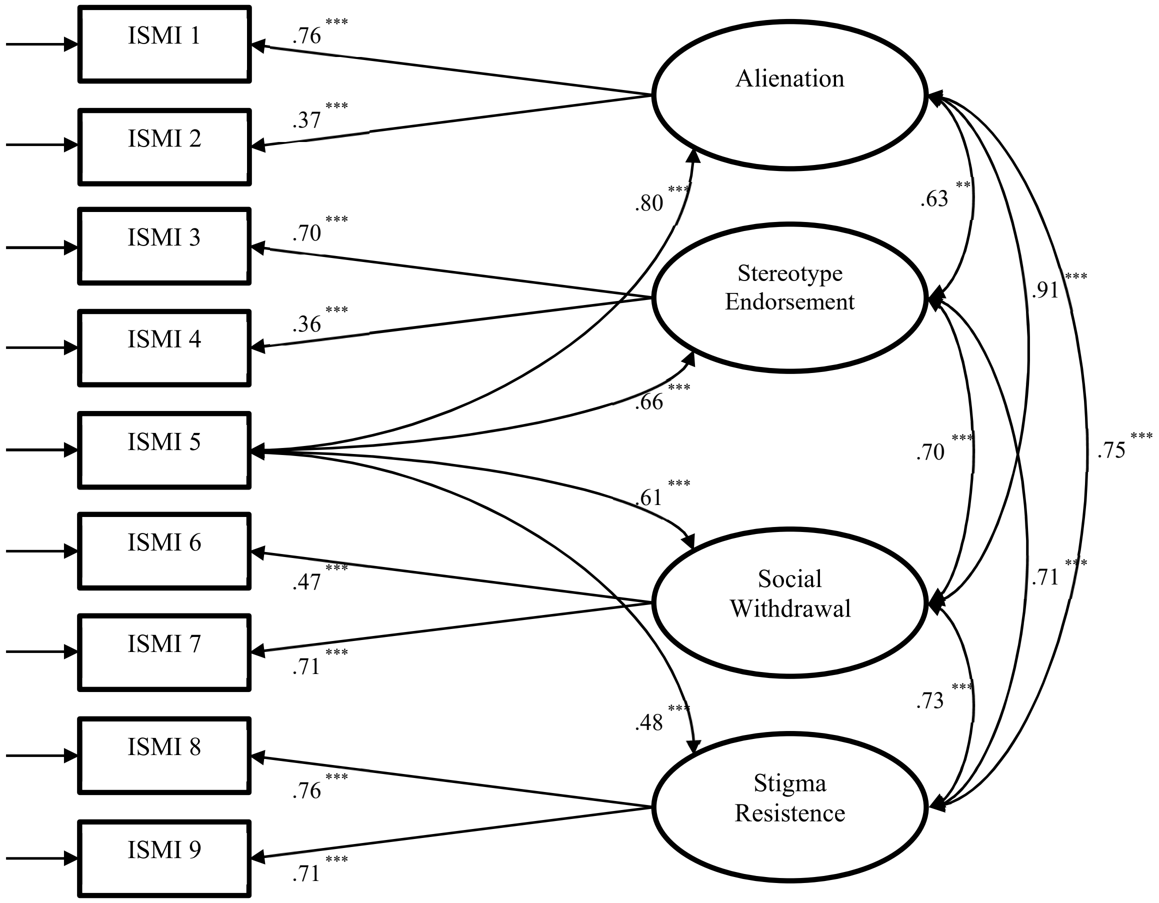

Supplementary Figure 1. ISMI-9 factor model with subscales
Note. Model fit: $\chi^2 = 31.47$, df = 18, $p = .025$, CFI = .97, RMSEA = .06, 90% KI [.02, .10], SRMR = .05.
$^{**}p < .01$, $^{***}p < .001$.

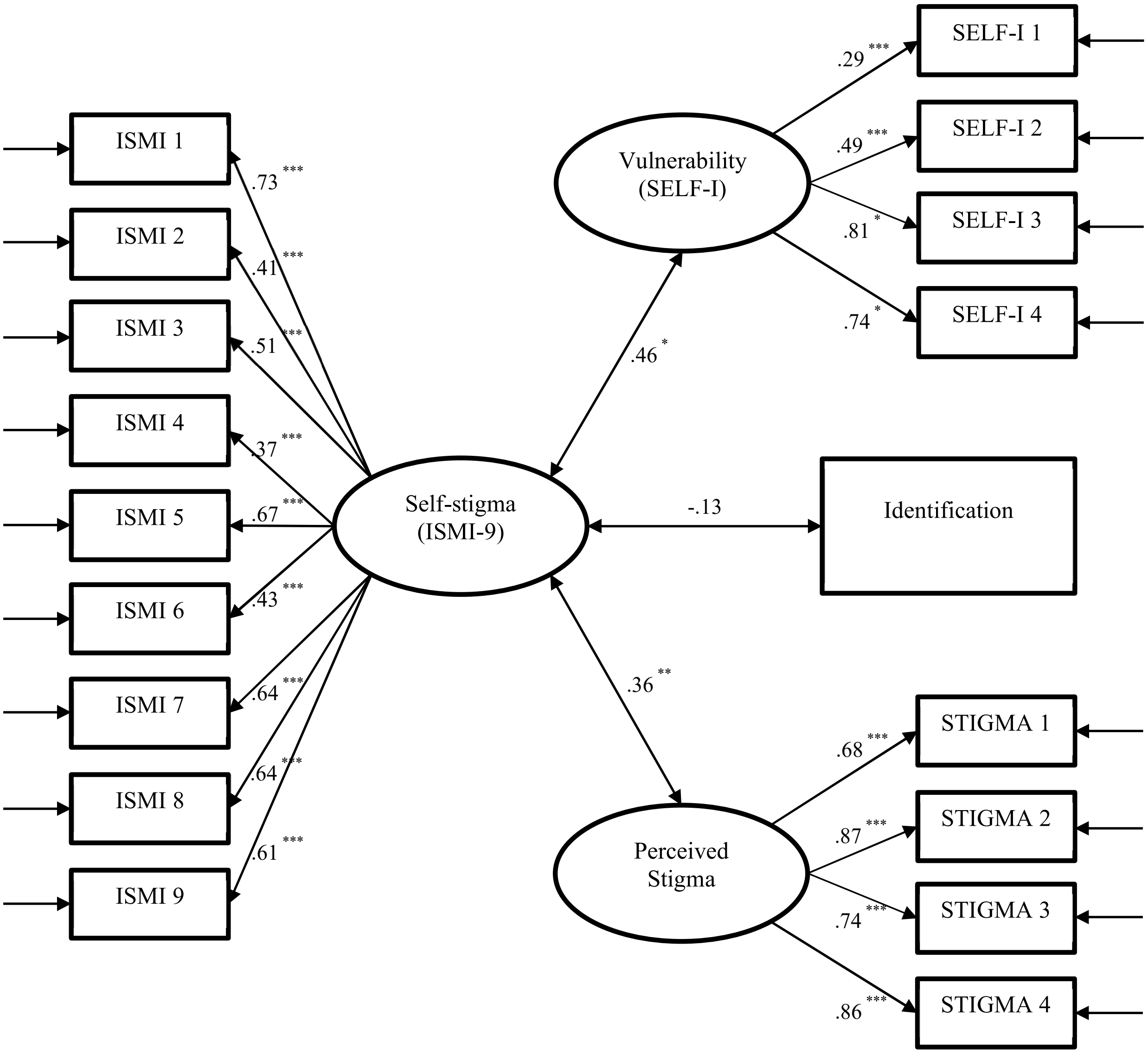

Supplementary Figure 2. Latent correlation analysis
Note. ISMI = Internalized Stigma of Mental Illness scale. SELF-I = Self-Identification of Mental Illness scale. Intercorrelations between perceived stigma, vulnerability, and identification were not statistically significant and thus omitted from the model. Model fit: $\chi^2 = 272.71$, df = 133, $p < .001$, CFI = .87, RMSEA = .08, 90% KI [.06, .09], SRMR = .08.
$^{*}p < .05$, $^{**}p < .01$, $^{***}p < .001$.
